# Mutual aid food sharing during the COVID-19 pandemic: case study of Tompkins County, NY

**DOI:** 10.1017/S1368980024001083

**Published:** 2024-10-21

**Authors:** Karla L Hanson, Sarah Coupal, Emily Grace, Elizabeth Jesch, Sonja Lockhart, Leah C Volpe

**Affiliations:** 1 Department of Public & Ecosystem Health, Cornell University, Ithaca, NY 14853, USA; 2 Division of Nutritional Sciences, Cornell University, Ithaca, NY, USA; 3 Mutual Aid Tompkins, Ithaca, NY, USA

**Keywords:** Mutual aid, Food security, COVID-19, Rural communities

## Abstract

**Objective:**

The COVID-19 pandemic led to greater food insecurity across the world, and government and charitable organisations did not always respond quickly enough or adequately to meet food needs. Mutual aid (MA) – neighbours helping neighbours to meet survival needs – mobilised residents to share food, often through outdoor food cabinets and refrigerators. This study aims to understand how MA food sharing was implemented, including food availability, acceptability, accessibility and impact on food access.

**Design:**

This case study describes one MA food sharing system by triangulating data from flyers, notes from nineteen volunteer meetings, six cabinet host interviews, data extracted from 1387 social media posts and 356 photographs, and 111 resident surveys.

**Setting:**

Tompkins County, NY, USA (total population about 100 000).

**Results:**

We estimated high availability of food: approximately 250 000 food servings were shared monthly, mostly carbohydrates. Most residents obtaining food found it acceptable, including satisfaction with food safety and cleanliness, food quantity, and ease of travel to the cabinets but were less satisfied with food variety. MA food sharing was accessible to food-insecure, unemployed and disabled residents, but not other priority populations. About two-thirds of residents reported improved food access. Volunteers exhibited tenacity and ingenuity in meeting operational challenges which included trash and vandalism, winter weather and unusable food contributions while foregrounding residents’ safety and privacy as shared values and navigating conflicting views about fairness.

**Conclusions:**

In times of crisis, MA can improve food access through free food sharing cabinets, but barriers include unacceptable food contributions and outdoor conditions.

The COVID-19 pandemic and concomitant economic losses negatively impacted food security around the world. During the first weeks of the COVID lockdown in the USA, food insecurity was estimated at 40 % or more of households in cross-sectional samples^(1–3)^ and double the expected rate in longitudinal data^(4)^. Local and regional samples suggested increases of 11–98 % depending on location and sampling methods^(5)^, with rural areas having worse food security than urban areas^(6)^. COVID-era data identified unemployed adults^(7)^, adults with fewer years of formal education^(2,3,8)^, adults who identified as Black, Indigenous, or other People of Color (BIPOC)^(2,3,9)^ and households with children^(2–4,8)^, as at greater risk of being food-insecure during this period.

In times of crisis and disaster, government and charitable organisations cannot always respond quickly or adequately enough^(10,11)^. Mutual aid (MA) is a grassroots response to immediate needs among community members. Through MA, community members ‘choose to help each other out, share things, and put time and resources into caring for the most vulnerable’^(10)^. This collective collaboration to meet the survival needs of all residents usually arises among members as ‘an awareness that the systems we have in place are not going to meet [survival needs]’^(10)^.

In the USA, MA has a long tradition in African-American, immigrant and anarchist communities^(10)^. In 2016, before the COVID-19 pandemic, Davies and colleagues^(12)^ identified over 4000 MA groups in a sample of 100 cities globally but noted that few of the initiatives provide any evidence to support claims of positive economic and social impacts^(12)^. Food sharing is a key component of MA systems although structures and processes vary, including: arranging food purchases and deliveries, preparing and distributing hot meals, partnering with local restaurants to donate meals and distributing grocery store gift cards^(11,13–18)^. One popular model uses a small structure similar to a ‘Little Free Library’ or refrigerator placed in a ‘community space in which people can give to, or take from, at any time, no questions asked’^(13,19)^. These structures are often outside and have been variously called food cabinets, blessing boxes, little free pantries, micro-pantries, community refrigerators or free fridges^(11,13,16,20)^. This model of food sharing is popular, with more than 200 pantries and refrigerators listed on the MA Hub website^(21)^.

In response to COVID-19, interest in MA food sharing grew substantially, new groups emerged and media coverage rose^(20)^. Recent studies have described MA systems developed in response to the COVID-19 pandemic in Cape Town, South Africa^(14)^; the United Kingdom (UK)^(18,22,23)^; Kenya, the occupied Palestine territory, the Philippines, and Sudan^(24)^; and nine cities in the USA^(15,20,25,26)^. We found no systematic data on MA systems in areas outside of cities. And, most of these studies do not systematically assess the scope of food sharing except a few broad estimates of food outputs. For example, MA groups in Cape Town reported providing food to ‘thousands’ of people^(14)^, and in the UK, twenty MA groups reported providing more than 100 000 meals and food packages^(18)^. Loften and colleagues^(15)^ describe three MA networks in Chicago, IL, that distributed 10 000 pounds of free food to more than 17 000 individuals in total^(15)^. These are some of the only quantitative data on outputs from MA systems in the USA. To the best of our knowledge, no one has estimated the impact of MA on residents’ food access or food security.

This case study focuses on a MA food sharing group operating in Tompkins County, NY. In response to the COVID-19 pandemic, this group assembled outdoor food sharing cabinets. The food cabinets are open 24 h every day and are stocked with free food and personal care items by volunteers. The MA Tompkins mission states: ‘We are prioritizing those most vulnerable and affected by COVID-19: the sick, elderly, disabled, undocumented, single parents, queer, Black, Indigenous, and/or people of color, those quarantined without pay, and those limited in work’^(27)^. Communication among community members flows through a social media group in which members post a cabinet location and photos of a full cabinet to alert others as to what is available or photos of an empty cabinet to show where items are needed at that time^(28)^. In November 2021, there were approximately 1500 members of this social media group.

MA has the potential to support the well-being of community members, increase food security and enhance public health, particularly in times of crisis. This case study seeks to describe how MA food sharing was implemented in one location outside of a major urban centre during the COVID-19 pandemic, explore food availability, acceptability and accessibility through MA food sharing and assess how MA food sharing impacted food access among food-insecure households. We adapted and applied Clay’s (2022) Disaster Food Security Framework (DFSF)^(29)^ and triangulated observational data with information reported from the perspectives of residents who obtained food and those who contributed food (groups that somewhat overlapped). The case study is unique in that it describes MA food sharing outside of the urban context, characterises the quantity and variety of food distributed using observational data and estimates the impact of food sharing on household food access.

## Methods

This descriptive case study was developed during more than 2 years of active participation by the authors in MA food sharing in Tompkins County. The case was selected as an instrumental example of a MA system of food sharing outside of a major city^(30)^. We strengthened the case study design by including analysis and synthesis of multiple data sources^(31)^. Triangulation of data from several sources allowed us to understand the operations of MA food sharing from the perspectives of residents who obtained food and those who volunteered as hosts or in other ways^(30,31)^. Additional methodological details are provided in the accompanying online supplementary material, Supplemental Materials. This research was exempted by the Cornell University IRB (#2108010508).

### Setting/Context

Tompkins County, NY, has approximately 100 000 residents spread over approximately 475 square miles^(32)^. Ithaca, the county seat and only city, has a population of approximately 31 000 and is classified as a small metropolitan area with substantial inbound commuting^(33)^. Surrounding Ithaca are small towns and farmland. Tompkins County is home to three colleges/universities with combined enrolment of more than 30 000 students: 28 % of the population are aged 18–24 years^(34)^. Median income in the County is $61 361, and 12·4 % of residents live in poverty^(32)^. Notably, more than half of adult residents (25 years and older) hold at least an undergraduate degree (53 %)^(32)^, compared with 38 % of the national population^(35)^.

### Data sources and analysis

Data for development of this case study came from the perspectives of residents in three MA roles: (1) ‘residents who obtained food’; (2) ‘hosts’ (residents who lead the operations and maintenance of a food sharing cabinet); and (3) ‘other volunteers’ (residents who contribute food or other resources, or help with maintenance, cleaning, or repairs). Five data sources were gathered between April 2020 and March 2022.

(1) MA Tompkins website: Maps on the website indicated the location of food sharing cabinets and were updated thirty-three times between May 2020 and November 2021.

(2) Monthly volunteer meetings: Hosts and other volunteers met monthly to discuss cabinet operations, address challenges and plan. Between July 2020 and March 2022, written notes were taken by a volunteer at nineteen meetings. These notes were inductively coded by the lead author using a descriptive coding technique to identify recurrent challenges and opportunities or proposed solutions^(36)^.

(3) Host interviews: Semi-structured interviews were conducted with cabinet hosts (a sub-set of volunteers) about their experiences and perceptions regarding MA food sharing. Hosts were recruited for interview through the public survey (described below) and announcements at two volunteer meetings. Interviewees provided verbal consent. Interviews were transcribed verbatim and inductively coded using a descriptive coding technique to describe their perceptions of MA food sharing and its strengths and weaknesses, and the operations and practices they used to manage the cabinet and its food donations^(36)^. Two authors independently coded all interview transcripts in NVivo (QSR International, version 12, 2020) with differences in coding resolved by consensus.

(4) Social media posts and photo documentation: Data were extracted from all photographs and comments posted to the MA food sharing social media group between September 2020 and February 2021 (*n* 1387). Residents obtaining food, hosts and other volunteers all contributed social media posts. To validate data extracted from social media posts and to provide data for some cabinets with little social media presence, sixteen cabinets were purposively selected for systematic longitudinal observation between January and February 2021^(36)^. Researchers took repeat photographs of cabinet and cooler contents from 09.00–19.00 hourly for 3 consecutive days within the City of Ithaca, and every 2 h for 5 d outside the City (*n* 356).

Data extracted from social media posts and photographs included location, date, time and the visible quantity of ninety-four different foods in various forms and sizes. For example, fruit was recorded as: fresh, piece (e.g. 1 apple); can/bag, medium (e.g. 20 oz can pineapple); and can/cup, individual serving (e.g. 3 oz mandarin oranges). For each food, servings were calculated in thirteen categories – meat, beans, eggs, nuts/nut butters, fruit juice, fruit, vegetables, dairy products, carbohydrates (rice, pasta and bread), prepared meals (sandwiches, soups and stews), sweets (soda, desserts, jam and candy), snacks (crackers, chips and granola bars) and added fats (butter, oil) – using the number of servings listed on the nutrition label for a frequently donated product in each category. Servings of meat, beans, eggs and nuts/nut butters were subsequently combined into servings of ‘protein foods’, and all categories were combined into total servings. Sums of servings were calculated in SPSS v.25 (IBM Corp.).

(5) Survey: A public survey assessed reach, satisfaction and perceived impact of MA food sharing. A convenience sample of residents was recruited in September–November 2021 through the social media group, volunteer listserv and flyers placed in the cabinets themselves. The survey utilised passive consent. An online survey asked about respondents’ role(s) in MA, their experiences and satisfaction, and demographic characteristics including race and ethnicity^(37)^, gender/sexual identity^(37,38)^, household composition, and the validated two-item food security screener^(39)^. Respondents who indicated that they obtained food from the cabinets were also asked their perceptions of the impact of the food sharing cabinets on their household food access and two open-ended questions about what was easy (difficult) about using the food cabinets. Respondents who obtained food from the cabinets were also offered pre-paid postcard surveys which asked only about satisfaction and perceived impact on food access and were available in each cabinet. Respondents who indicated they hosted a cabinet or volunteered in another way were asked two open-ended questions about what was easy (difficult) about supporting the food cabinets. Respondents who both obtained food and hosted or volunteered were asked both sets of questions. Survey data were summarised with counts and percentages in SPSS v.25 (IBM Corp.). Responses to open-ended survey questions were hand-coded into emergent ‘easy’ and ‘difficult’ attributes of the food sharing cabinets by one author.

### Triangulation and synthesis

‘Member checks’ in which findings are discussed with respondents to validate or verify the information, to maintain transparency and trust, and enhance construct validity were performed during MA volunteer meetings after analysis of each type of data^(31)^. We adapted the DFSF framework^(29)^ to consider availability (cabinet locations and total contributions), acceptability (safety and cleanliness, nutrition, and needs and preferences) and accessibility (physical, economic and social). Each dimension was assessed by multiple data sources (see Table 1). Data were not available to assess residents’ agency with respect to food security. Data were triangulated by considering both compatible and contradictory information, assessed for importance and completeness, and synthesised and used thematically to draft this descriptive case study^(31)^.

**Table 1:** Data sources describing each disaster food security dimension

Dimension	Data source					
	MA website	Volunteer meeting notes	Host interviews	Social media posts and photo documentation	Public survey	
					Residents who obtained food	Hosts and other volunteers
Availability	• Flyers		• Sources of contributed food	• Total contributions		• Food contributions • Satisfaction with contributions
Acceptability						
• Safety and cleanliness		• Barriers to food safety and cabinet cleanliness • Proposed solutions	• Perceptions of food safety and cabinet cleanliness		• Satisfaction with food safety and cabinet cleanliness	
• Nutrition				• Contributions by food category	• Satisfaction with food quantity and variety	
• Needs and preferences			• Descriptions of food donations	• Commonly contributed food items	• Satisfaction with meeting special diet needs and food requests	
Accessibility						
• Physical		• Barriers to physical accessibility • Proposed solutions	• Perceptions of cabinet accessibility		• Pattern of cabinet use • Satisfaction with ease of travel to cabinets	
• Economic					• Food insecurity • Use of food assistance • Perceived change in food access	
• Social			• Perceptions of fairness and privacy		• Reach to priority populations	

## Results

### Sample descriptions

No characteristics were recorded for residents who participated in volunteer meetings or posted on social media. There were 111 responses to the survey: fifty-seven respondents obtained food (including twenty-one postcards) and eighty-four volunteered (including hosts). Thirty respondents both obtained food and volunteered, were asked questions about both activities and were included in both samples. Six cabinet hosts were interviewed: two within the City of Ithaca and four outside the City.

### Availability

**Fig. 1:**
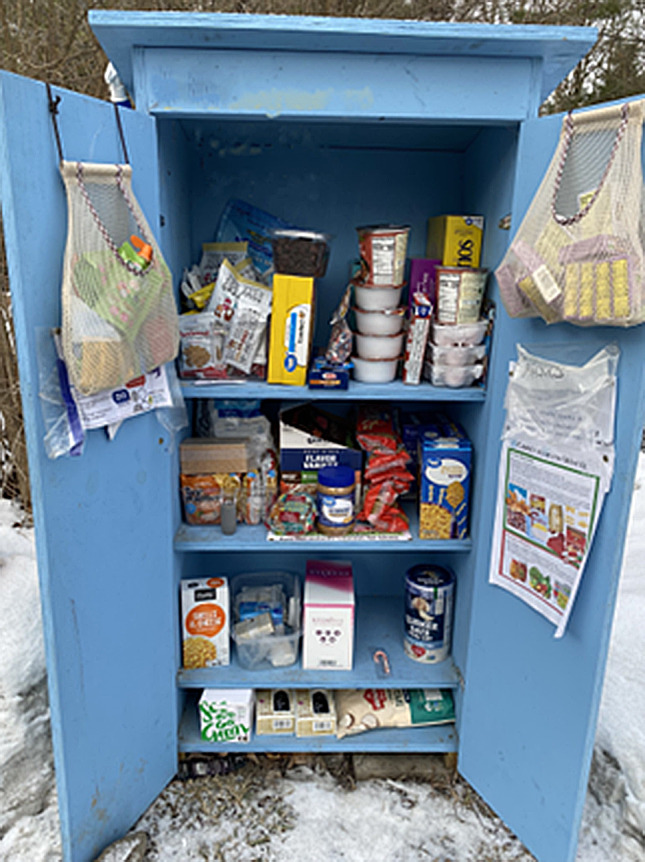
Mutual aid food sharing cabinet

The website and volunteer meeting minutes described how the food sharing cabinets were built by volunteers with plywood, roof shingles and other materials purchased with cash donations (see Fig. 1). The cabinets were designed to stand outdoors through all four seasons in upstate NY. A few cabinets included coolers for fresh produce and dairy products, and two had adjacent indoor refrigerators.

**Fig. 2:**
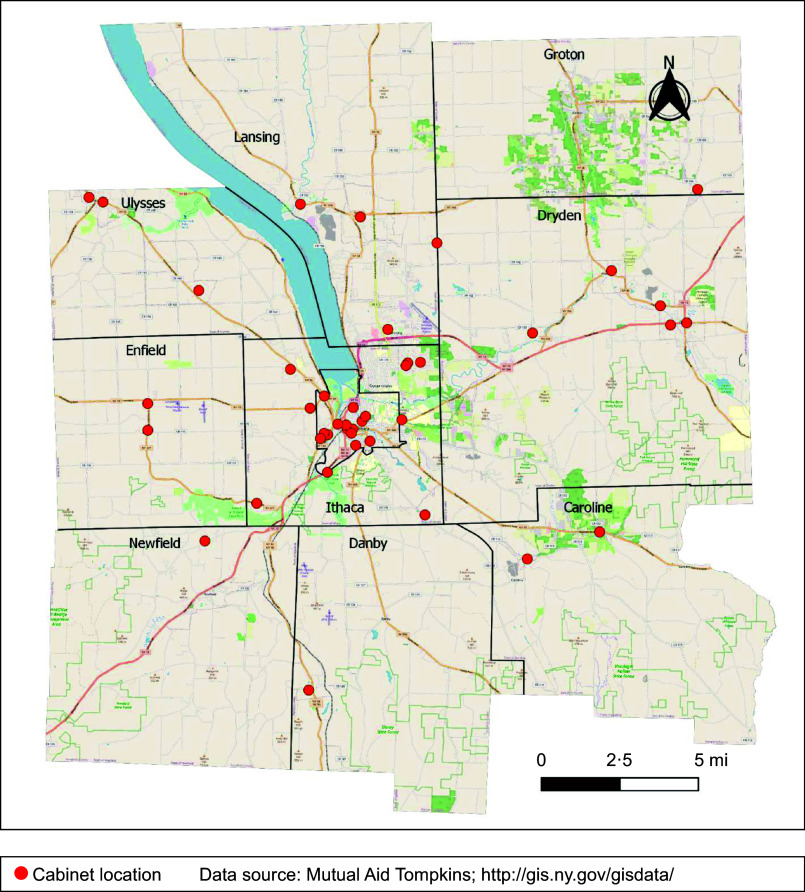
Mutual aid food sharing cabinet locations, Tompkins County NY – January 2021

In January 2021, the website indicated forty-three food cabinets positioned throughout the County including many in the City of Ithaca (see Fig. 2). Locations were listed on a flyer distributed throughout the county and also were mapped on the MA Tompkins website^(27)^. Some cabinet locations aligned with populations prioritised in the mission statement: ten supported or lower-rent housing complexes, seven non-profits (two of which specifically served BIPOC residents), four churches, three schools and three community centres, with the remainder mostly located outside private homes. Two additional cabinets were located near encampments of unhoused residents but were not listed on the website or surveyed for the privacy and safety of the residents. Although the total number of cabinets remained somewhat constant (range, 38–51), by November 2021, only eighteen remained in their May 2020 locations.

Most surveyed volunteers reported spending their own money (71·4 %) and less than 1 h per week (53·2 %) delivering 1–3 grocery bags of food and supplies to the cabinets (47·6 %). Most volunteers were satisfied with the amount of time and money they spent supporting MA food sharing (76·1 and 64·4 %, respectively). Every host interviewed described unique arrangements through which they obtained food and other supplies for the cabinet, including stores, church and school groups, a local food rescue non-profit organisation, and other sources. Hosts also observed that some donated foods appeared to originate from the food bank because they ‘recognise all of the usual things’ and noted an ‘overflow of pasta and peanut butter’ in the cabinets. However, the origin of many contributions was not known.

‘The only one I know helps fill the box is [a neighbor]. They live near me. Everyone else who donates, I don’t know them… I keep my distance, so I haven’t met anyone else who donates’. (host interview 1, outside city)

### Acceptability – food safety and cleanliness

Several challenges to cleaning cabinets and ensuring safe, high-quality foods emerged from both volunteer meeting notes and host interviews. A substantial amount of time at volunteer meetings was spent discussing trash, vandalism and neglect at some cabinets, and how to address those issues or whether to move the unclean cabinets. One host described how one unsightly cabinet reflected on the MA food sharing programme overall.

‘I come down [the street] and there’s a [cabinet] that, it’s just basically, like, trashed and usually empty and sort of like open and not very appealing anymore. I think it just is sort of, like, forgotten. Those forgotten ones or those sort of, like, yucky looking ones detract from the ones that are working, I think… I would think about either moving or, like, sprucing up or reassigning some of those cabinets that have been not getting as much maintenance all along’. (host interview 6, city)

In discussing this cabinet, volunteer meeting minutes showed concern about cleanliness and safety balanced with interest in meeting the needs of the community members it served who (given its location) were likely to have issues like addiction and mental health, or to be unhoused. Despite concerns about the cabinet’s unsightliness, the volunteers decide it was important to leave the cabinet in place.

The most intractable challenge seemed to be dealing with ‘past-date’ and spoiled food and related trash. This host described their concerns about such donations,

‘I’ve had some real problems with people bringing foods that are way out of date. We just had somebody come and share food, 2016 it was labeled! So, we are carefully pulling those out and we get a lot of that… I’m nervous about that because people are trusting what we’re placing in that blue cabinet. I don’t want anybody getting sick’. (host interview 3, outside city)

Minutes recorded one meeting at which MA volunteers examine foodbank guidelines for package dates and safe food consumption^(40,41)^. These guides explained the difference between expiration dates (beyond which foods like meat or milk may be unsafe to consume) and ‘use by’ dates (beyond which shelf-stable foods like dried and canned items may not be at peak freshness). Further, one guide recommended that shelf-stable foods can be safely consumed for 1–3 years after the use-by date depending on the product^(41)^. However, MA volunteers came to consensus that no ‘past-date’ foods should be shared via the cabinets. Instead, they opted to use the social media group to ‘encourage people to add the type and quality of items that they themselves would like to eat’.

A related challenge was contributions of perishable food that spoiled before being taken. Volunteers described needing to clean up ‘big messes’, ‘bring home compost and trash’ and bring ‘past-date’ foods to a local pig farmer. Volunteers described it as ‘tough to deal with’, ‘disheartening’ and ‘depressing’. These two hosts described the impact that spoiled food had on their cabinet operations.

‘So, I came home at 11 o’clock at night to find like huge boxes … This was, like, last year when [the food bank] kept giving out those sausage patties to everyone, that everyone was getting sick of… This was summer high heat like 80, 90-degree days and I had boxes and boxes of these sausage patties sitting in front of my house mixed in with a bunch of can donations that were getting ruined because the sausage patties were, like, laying on top of them. And I’m like, ‘Who does that?’ you know’. (host interview 1, outside city)

The efforts of the cabinet volunteers appeared to be successful because approximately three-quarters of surveyed residents who obtained food were satisfied with the safety of foods available (75·5 %) and the cleanliness of the cabinets (71·7 %).

### Acceptability – nutrition

Data extracted from photo documentation estimated that 266 000 servings of food were shared each month. Carbohydrates were the largest food category (94 435 servings/month), comprising approximately one-third of all food shared. Protein foods, vegetables, fruits and juices, snacks, and sweets were all estimated at 25 000–30 000 servings/month. Estimates from social media posts were much lower than photo documentation (see Table 2).

**Table 2:** Estimated food servings distributed per month

	Serving examples	Photo documentation	Social media posts
Total servings per month		266 335	23 797
Servings by category:			
Total carbohydrates	2 oz. rice/pasta, 1 slice bread	94 435	8132
Total protein	2–3 oz. meat, 4 oz. beans, 1 egg, 1·5 oz. nuts	30 402	3117
Total vegetables	1c. or item	29 254	3148
Total fruit and juice	4 oz. or 1 item fruit, 6 oz juice	28 332	1826
Total snacks	0·5 oz. crackers, 1 oz. chips, 1 granola bar	27 390	2409
Total sweets	8 oz. soda, 1 cookie or slice of pie, 1 oz. candy	25 903	1969
Total dairy products	8 oz. milk or yogurt, 1·5 oz. cheese	22 365	1020
Prepared meals and sandwiches	1 sandwich	7802	692
Total added fats	1 Tbs. oil or butter	5432	381

Approximately half of surveyed residents who obtained food were satisfied with the quantity of food available from the cabinets (52·8 %), and few reported rarely or never finding food in the cabinet (19·4 %). However, several survey comments suggested that timing was important; for example, one resident commented that ‘Sometimes I arrive an hour after knowing [the cabinet] is full and it’s already been emptied’. Some survey respondents who obtained food were satisfied with the variety of foods offered (39·6 %), but others requested changes such as more inventory and more ‘fresh, healthy, natural, and vegetarian foods’.

### Acceptability – needs and preferences

In interviews, cabinet hosts described donating ‘ready-to-eat staples’ like ‘microwave rice dishes’ and ‘stuff you could eat right out of the bag’ like ‘granola bars’. As one host described, ‘[I] get, like, thinking about people at home with their kids and, like, what’s easy for people to make. What can younger people prepare at home’ (host interview 2, outside city). Photo documentation suggested that the ten most frequently contributed food items and sizes were primarily ready-to-eat and easy-to-prepare items: single-serving canned fruit, oatmeal, chips/pretzels, and granola bars; fresh fruit/vegetables; mac-and-cheese and other pasta; prepared meals (e.g. stews, sandwiches, curries); and tomato sauce.

Eighteen survey respondents reported that someone in their household ate a special diet, including dairy-free (6), vegan or gluten-free (5), vegetarian or low-carb (4), peanut-free or tree nut-free (3), and egg-free (2). Volunteers experimented with multiple mechanisms for accepting food requests at the cabinets: dry-erase boards, notepads and chalk boards with some success. One cabinet host described fulfilling these requests.

‘There are two people that I still purchase things for… They’re gluten-free, and had two kids and they had a lot of dietary restrictions… I did some specific shopping for them and, you know, it turns out in the end I just give them gift cards and let them go get what they need. You know, because I’m not gluten free, I don’t know what they need’. (host interview 2, outside city)

Nine of the survey respondents in households with special diet needs reported that cabinet foods met their needs (50·0 %), and twelve who made a special request were satisfied with how it was addressed (75·0 %).

### Accessibility – physical

Survey respondents most often reported obtaining food from a cabinet 2–3 times a month (32·4 %) or 1–2 times per week (26·5 %), and taking 1–3 food items each time they visited a food sharing cabinet (71·9 %). Most were satisfied with the ease of travel to the cabinets (81·1 %). They described the cabinets as convenient because they were noticeable, within walking distance or close by, accessible 24 h/d, and that many had parking. A few survey respondents noted that the cabinets were difficult to access because of a disability.

Interviews with cabinet hosts illustrated how the accessibility of the food cabinets also made it easier for cabinets to be maintained and for contributions to be made. Some noted locations that are ‘all over’ and ‘so convenient to stop at on the way home from shopping’. Others appreciated having 24-h access to the cabinets and ‘being able to work on the cabinets any time at all is perfect; minimal coordination is awesome!’

Although many of the operational characteristics supported accessibility, volunteer meeting notes indicated that coping with weather was a big challenge to outdoor food sharing cabinets, particularly during winter months with freezing temperatures and a total of more than 60 inches of snow per year. There was also a need to protect food – particularly produce, jars and cans – from freezing temperatures. Volunteers developed a flyer describing which foods could be safely contributed during winter and hung it in all the cabinets. Volunteers also experimented with insulating foods in coolers. Many MA members took in perishables and breakables during freezing temperatures and returned them to a cabinet when the weather warmed. One cabinet had access to electricity and the host purchased a warming plate designed for egg incubation to heat the cabinet.

‘[The warming plate] is this weird flat black thing. It’s only like three quarters of an inch thick… It keeps things warm to about minus 10. If it gets below minus 10, we need to bring things in, but that’s not that often… So, it’s a little different [than other cabinets] because the bottom shelves [where the warmer is], at least, can be cans and jars and whatever all winter’. (host interview 5, outside city)

Volunteer meeting notes documented that a few cabinets closed for the winter due to volunteer capacity, financial strain and weatherisation issues. A few other cabinets had to be moved so that they would not be knocked over by plowed snow. Most cabinets needed to be shoveled out, which was accomplished through direct requests or via social media rather than coordinating centrally. One cabinet host explained,

‘Last winter there was a time we got this major big storm. Then, plows on my little road kept plowing so the cabinet was completely covered. You could only see its roof. And, so, I took a picture of it, and I posted it [to social media group]… saying “Anyone want to come help shovel?” A neighbor who also does a lot of volunteer work for mutual aid, came with a plow truck and shovels and got it all cleaned out really nice’. (host interview 1, outside city)

### Accessibility – economic

Almost all online survey respondents who obtained food lived in a food-insecure household in the month of the survey (81·5 %), compared with 11·6 % of adults in the prior year in the county overall^(42)^. Most households had monthly income of $3000 or less (85·2 %). Most also had other help obtaining food (81·5 %), most often SNAP (63·0 %), food pantries (37·0 %) and free/reduced-price school meals for their children (22·2 %). The majority thought that their food access had improved since obtaining food from the cabinets (64·2 %).

### Accessibility – social

The food sharing cabinets reached some MA priority populations more than others (see Table 3). Among online survey participants, disabled adults and households experiencing unemployment appear more often than rates in the county overall. Conversely, only four surveyed residents who obtained food identified as BIPOC (15·4 %), whereas 23 % of County residents could be categorised as BIPOC.

**Table 3:** Reach to priority populations among online survey respondents who obtained food

Priority population	Respondents who obtained food* (*n* 26)		Tompkins County	
	Count	%		%
Respondent identified as:				
BIPOC	4	15·4		22·6^(32)^
LGBTQ	7	31·8		Unavailable
Household included:				
Seniors (65+ years)	5	19·2		15·1^(32)^
Disabled adult(s)	11	42·3		7·3†^(32)^
Adults unemployed, past month	17	65·4	Peak:‡	11·3^(44)^
			During survey:§	3·0^(44)^
Single-parent household	4	15·4		33·3||^(45)^

BIPOC, Black, Indigenous, or other People of Color.

* Does not include postcard respondents.

† Percentage disabled in population < 65 years.

‡ Peak unemployment rate for adults during study period (April 2020).

§ Unemployment rate at the time of the survey (October 2021).

|| Percentage of children in single-parent household.

Conflicting views about fairness was a recurrent challenge and emerged from both volunteer meeting notes and host interviews. Some believed that people know what they need and should not be judged, as this host explains.

‘I am not on the list of “concerned about people double dipping”. Oh my goodness! Imagine that, getting an extra loaf of bread. I don’t subscribe to that fear…I’m not going to penalize everybody else because they go to their local food pantry but can’t get enough food to feed their children. I’m not going to tell them “No, you can’t get food from us.”’ (host interview 3, outside city)

Others were concerned that one person could take everything out of a cabinet leaving nothing for others, and they took actions to prevent this from happening.

‘I keep spare of everything that I put in the blue cabinet in the trunk of my car so that, like, I only put out, like, one or two items, similar items. Like one or two hamburger helpers and a taco kit or something like that, rather than like keeping the cabinet full of them… When I first got started, I know that I, like, if I bought nine boxes of taco makings, I would put them all in the cabinet and then the next day they’d all be gone… that’s when I started, like, rationing out and putting, like, one or two of each item at a time out there and then checking it daily… If I see someone come and I believe it might be a donation, I’ll go look and if they put like 20 bags of mashed potato mix in there, I’ll take most of them out, leave like two and keep them in my trunk and put them back in one or two at a time, just so that that wouldn’t happen’. (host interview 1, outside city)

Volunteer meeting notes described brainstorming the ways they view MA food sharing as different from food assistance or food charity. Volunteers agreed that the operational characteristics of the food cabinets, such as the open-access model, offered anonymity to those who wanted it. This theme was also present in host interviews, including one cabinet host who described how they identified with the need for help with food at one point in their life, how they desired privacy when seeking help and how the cabinets are uniquely positioned to meet this need.

‘I think a big part is that there’s less stigma around going to a cabinet. So instead of, like, going somewhere and saying ‘Hi, I need help, I don’t have food’, is different than ‘Okay, this cabinet has bread and I really need bread’, and just being able to stop by. I know that that’s been a barrier for me. It’s difficult to admit that I need food assistance’. (host interview 4, city)

## Discussion

This case study provides in-depth description of one COVID-era MA food sharing group outside of an urban context. The emergence of a network of about 50 food sharing cabinets in Tompkins County was a substantial change to the *availability* of free food locally. Observational data estimated approximately 250 000 servings/month of free food were distributed. However, social media posts showed that less food was shared than did our systematic photo documentation, suggesting that contributors do not always post contributions on social media. Because many COVID-era MA groups relied on social media for communication^(28)^, it is important to note that social media may underestimate the breadth of MA food sharing.

Overall, residents mostly found MA food sharing *acceptable*, although volunteers described multiple challenges to providing safe and nutritious foods via outdoor food sharing cabinets. Residents who obtained food were generally satisfied with the cleanliness and safety of the food sharing cabinets. These findings align with another study that described micro-pantries and found that residents who obtained food had little concern over food safety^(20)^. However, volunteers continually grappled with donations of past-date and spoiled foods and trash which threatened food safety and cleanliness. Volunteers crafted solutions which balanced safety and cleanliness with community needs. For example, volunteers remained steadfast in prohibiting out-of-date foods from the cabinets even when edible by published standards^(40,41)^ and in retaining cabinet locations that served particularly vulnerable populations even if they were ‘trashed’ and required more extensive upkeep.

Residents who obtained food found the quantity of food in the MA cabinets acceptable, but the variety of foods lacking. The DFSF emphasises the need for prepared or ready-to-eat food during times of disaster^(29)^, so the frequent contribution of these items was likely advantageous. However, one-third of all food servings were carbohydrates which suggests that the variety of foods may not meet nutritional needs. Furthermore, the weather challenges reported by volunteers suggest that food sharing may be limited in its ability to expand variety to include fresh foods (that need to be kept cool in summer weather) and canned foods (that need to be protected from freezing in winter weather) until cabinet locations with consistent temperatures can be secured.

The MA food sharing cabinets were physically *accessible* to residents when obtaining or contributing food and when providing maintenance. The cabinets reached food-insecure residents, many of whom remained food-insecure despite accessing government food assistance and/or food charity. And importantly, most residents reported that their food access improved since they began getting food from the cabinets. To our knowledge, these are the first data to empirically document the impact of MA food sharing on food access.

Despite strategic placement of cabinets, our data suggest that MA food sharing was not accessible to all priority populations. In particular, survey data suggest that MA food sharing did not adequately reach community members who identified as BIPOC. However, it is unclear how low survey participation among this group may have influenced our estimates of reach to BIPOC residents^(43)^. There are also incomplete local data to which we can compare reach to LGBTQ community residents and assess its adequacy. Further research is needed to better understand how food sharing could better reach these priority populations.

This case described one example of a MA system of food sharing outside of a major city and provided a rich exploration of this phenomenon in its natural context ^(30)^. Strengths of this case study include long-term involvement in MA Tompkins by several authors, triangulation and synthesis of multiple data sources to develop a complete picture^(31)^ and observed consistency between some elements of this case and several MA systems in other locations^(15,18,22,26)^.

The case study had several limitations that deserve note. First, survey results were limited by small sample sizes relative to County population and the geographic breadth of the cabinets. Second, some residents contributed data in more than one MA role (e.g. as a volunteer recorded in meeting notes and as an interviewed host), and their perspectives may be overrepresented in the results. Third, the results of this case study may not be generalisable to systems of MA in other locations or time periods. Tompkins County is unique in its density of young adults (28 % of the population are aged 18–24)^(34)^ and educated residents (53 % hold at least an undergraduate degree)^(32)^. Furthermore, this sytem of MA food sharing emerged during the COVID-19 pandemic which also permanently changed institutions, systems and technologies. These findings may not be generalisable to future MA food sharing in an evolving landscape.

This case study provides novel and important evidence regarding the availability, acceptability and accessibility of one local MA system of food sharing across a county that includes rural areas. Data suggest successful distribution of acceptable and accessible food to meet survival needs, and consequent improvements in food access among food-insecure residents. Furthermore, the case study identified substantial barriers posed by unacceptable food contributions and outdoor conditions. MA has the potential to support the well-being of community members through increased food security during health and economic crises.

## Supporting information

Hanson et al. supplementary material Hanson et al. supplementary material

## Supplementary material

For supplementary material accompanying this paper visit https://doi.org/10.1017/S1368980024001083
