# Supplementary material for: Mutual aid food sharing during the COVID-19 pandemic: case study of Tompkins County, NY
Source: Public Health Nutr. 2024 Oct 21;27(1):e215. doi: 10.1017/S1368980024001083 (PMC11604318; doi:10.1017/S1368980024001083)
Supplement: Hanson et al. supplementary material [file S1368980024001083sup001.docx]

Supplemental Materials

Mutual aid food sharing during the COVID-19 pandemic: Case study of Tompkins County, NY

**ADDITIONAL DetailS on Study Methods**

Photo documentation: Sixteen cabinets were selected for systematic observation to validate data extracted from social media posts and to provide data for some cabinets with little social media presence. Six cabinet locations had 50 or more social media posts (five within and one outside Ithaca) and were selected for systematic observation. Ten additional locations (two within and eight outside Ithaca) were selected from across the County to represent most areas where MA food sharing cabinets were located (Caroline, Dryden, Enfield, Freeville, Lansing, Newfield, and Trumansburg). In January-February 2021, researchers took repeat photographs of cabinet and cooler contents and recorded the location, date, and time from 9am to7pm. Cabinets within Ithaca were photographed hourly for 3 consecutive days, and cabinets located in less populated areas outside Ithaca were photographed every 2 hours for 5 days. Data were extracted from these photographs as described above.

Data Extraction from Social Media Posts and Photographs: Data were extracted from all social media group photographs and comments between September 2020 and February 2021, and included location, date, time, and the visible quantity of 94 different foods in various forms and sizes. For example, fruit was recorded as: fresh, piece (e.g., 1 apple); can/bag, medium (e.g., 20oz can pineapple); can/cup, individual serving (e.g., 3oz mandarin oranges); dried, individual serving (e.g., mini box raisins); dried, medium (e.g., 20oz box raisins); or jar, medium (e.g., 24oz jar applesauce). A photograph of a completely empty cabinet would be coded as zero for each food item. Data also were extracted from comments about the cabinet *before* the photo (e.g., “Only two cans of beans when I got here”), and *afterwards* (e.g., “By 6pm everything was gone except one bottle of water”).

Calculation of Food servings: Both social media and photo data were subsequently recoded into food servings in 13 categories -- meat, beans, eggs, nuts/nut butters, fruit juice, fruit, vegetables, dairy, carbohydrates (rice, pasta, bread), prepared meals (sandwiches, soups, stews), sweets (soda, desserts, jam, candy), snacks (crackers, chips, granola bars), and added fats (butter, oil) -- using the number of servings listed on the nutrition label for a frequently donated product in each category. Servings of meat, beans, eggs, and nuts/nut butters were subsequently combined into servings of “protein foods,” and all categories were combined into total servings.

Extracted data may have been biased because volunteers sometimes moved foods out of the cabinets, either to find more favorable locations for the items or to remove a particularly unpopular food. Based on discussions in volunteer meetings, movement between cabinets happened most often with canned beans and soups, and dried beans were usually removed from the cabinets because they were seldom taken. To account for this potential for over-estimation of food servings distributed through the cabinets, estimated servings of canned beans and soups were reduced by 50% and dried beans were set to zero.

Public survey: Respondents who indicated that they obtained food from the MA Tompkins food sharing cabinet were asked closed-ended questions to assess for the prior month: the frequency with which they visited cabinets (7 response options: 1 time to multiple times/day), how often the cabinets had food available when they visited (5 response options: never to always), and the number of food items they typically obtained each visit (4 response options: 1-3 items to 10+ items). Impact was assessed by asking, “Since you started getting food from the food sharing cabinets, how has your access to food changed?” (5 response options: improved to decreased). Five dimensions of satisfaction (ease of travel to the cabinet, cleanliness, food safety, quantity of food, and variety of foods) were assessed for the respondent’s most frequently visited cabinet location (5 response options: very satisfied to very dissatisfied). These respondents were also asked if anyone in their household followed a special diet and were provided with 10 restricted diet options as well as a text box. Respondents were also asked whether they had ever made a special food request. Respondents who indicated a special diet were asked about their satisfaction with the availability of food that met their family’s special diet needs, and respondents who has made a special request were asked about their satisfaction with how the request was addressed. Finally, open-ended questions asked respondents to describe anything easy (and difficult) about using the food sharing cabinets.

Respondents who indicated that they shared food, hosted a cabinet, or volunteered another way were asked closed-ended questions to assess for a typical week: how many hours were spent supporting MA food sharing (6 response options: 1 hour to 20+ hours/week), how many grocery bags of food and other items were delivered (numeric response), and whether they spent their own money supporting MA food sharing. Three dimensions of satisfaction (time spent, money spent, communication) were assessed (5 response options: very satisfied to very dissatisfied). These respondents were also asked if they think there are enough volunteers. Finally, open-ended questions asked these respondents to describe anything that is easy (and difficult) about volunteering with MA Tompkins.

The on-line survey included seven questions to assess whether respondents identified with MA Tompkins priority populations. Races and ethnicities were recorded as American Indian or Alaska Native; Asian or Asian American; Black or African American; Hispanic, Latinx, or Spanish origin; Middle Eastern or North African; Native Hawaiian or Pacific Islander; White; other race or ethnicity not listed above; and prefer not to answer.^(1)^ Respondents who indicated at least one identity other than white alone were counted as Black, Indigenous, or people of color (BIPOC). Gender and sexual identities were recorded as: straight; gay or lesbian; bisexual; transgender, transsexual, or gender non-conforming; queer; none of the above; and prefer not to answer.^(1, 2)^ Respondents who indicated at least one identity other than straight alone were counted as Lesbian, Gay, Bisexual, Transgender, or Queer (LGBTQ). The survey asked how many adults were in the household, and how many of those adults were 65 or older, how many disabled, and whether any of the adults were unemployed in the past month. The survey also asked how many children were in the household, and households comprised of child(ren) and one adult were counted as single-parent households. Respondent households were also characterized as food secure or insecure in the past year using responses (often true, sometimes true, or never true) to two statements: ‘I worried whether our food would run out before we got money to buy more,’ and ‘The food that we bought just didn’t last and we didn’t have money to get more.’^(3)^ Respondents who indicated that either statement was sometimes or often true were counted as living in a food insecure household.

**REFERENCES**

1. Kaplowitz R, Laroche J. More than Numbers: A Guide Toward Diversity, Equity, and Inclusion (DEI) in Data Collection. Tulsa, OK: Charles and Lynn Schusterman Family Foundation; 2020.

2. The GenIUSS Group. Best Practices for Asking Questions to Identify Transgender and Other Gender Minority Respondents on Population-Based Surveys. Los Angeles, CA: The Williams Institute; 2014.

3. Gundersen C, Engelhard EE, Crumbaugh AS, et al. Brief assessment of food insecurity accurately identifies high-risk US adults. Public Health Nutr. 2017;20(8):1367-71.
